# MTDL-EPDCLD: A Multi-Task Deep-Learning-Based System for Enhanced Precision Detection and Diagnosis of Corn Leaf Diseases

**DOI:** 10.3390/plants12132433

**Published:** 2023-06-23

**Authors:** Dikang Dai, Peiwen Xia, Zeyang Zhu, Huilian Che

**Affiliations:** 1 School of Cyber Science and Engineering, Nanjing University of Science and Technology, Nanjing 210000, China; dikangdai@njust.edu.cn (D.D.); peiwenxia@njust.edu.cn (P.X.); 2 Beijing Advanced Innovation Center for Food Nutrition and Human Health, College of Food Science and Nutritional Engineering, China Agricultural University, Beijing 100083, China; 2017308010216@cau.edu.cn

**Keywords:** deep learning, corn leaf disease detection, multi-task system, mobile application, spatial attention mechanism

## Abstract

Corn leaf diseases lead to significant losses in agricultural production, posing challenges to global food security. Accurate and timely detection and diagnosis are crucial for implementing effective control measures. In this research, a multi-task deep learning-based system for enhanced precision detection and diagnosis of corn leaf diseases (MTDL-EPDCLD) is proposed to enhance the detection and diagnosis of corn leaf diseases, along with the development of a mobile application utilizing the Qt framework, which is a cross-platform software development framework. The system comprises Task 1 for rapid and accurate health status identification (RAHSI) and Task 2 for fine-grained disease classification with attention (FDCA). A shallow CNN-4 model with a spatial attention mechanism is developed for Task 1, achieving 98.73% accuracy in identifying healthy and diseased corn leaves. For Task 2, a customized MobileNetV3Large-Attention model is designed. It achieves a val_accuracy of 94.44%, and improvements of 4–8% in precision, recall, and F1 score from other mainstream deep learning models. Moreover, the model attains an area under the curve (AUC) of 0.9993, exhibiting an enhancement of 0.002–0.007 compared to other mainstream models. The MTDL-EPDCLD system provides an accurate and efficient tool for corn leaf disease detection and diagnosis, supporting informed decisions on disease management, increased crop yields, and improved food security. This research offers a promising solution for detecting and diagnosing corn leaf diseases, and its continued development and implementation may substantially impact agricultural practices and outcomes.

## 1. Introduction

Corn, as a pivotal agricultural crop, exerts a profound influence on the global food supply and is deeply intertwined with human daily life. This versatile plant serves as a fundamental source of sustenance for humans, a primary component of animal feed, and a key raw material for a myriad of industries. Owing to the extensive scale of production and the consequential prominence of corn, it is indispensable for maintaining agricultural development and guaranteeing food security on a global scale.

Corn leaf diseases represent a substantial risk to crop yields, potentially resulting in considerable reductions in agricultural productivity. Among the multitude of diseases impacting corn foliage, Blight, Common Rust (Puccinia sorghi), and Gray Leaf Spot (Cercospora zeae-maydis) are especially detrimental, inflicting significant damage on crop growth and yield. Given the deleterious consequences of these diseases on corn production, the development of efficacious approaches for the detection and diagnosis of corn leaf diseases is of the utmost importance.

The identification of corn leaf diseases presents a formidable challenge, as numerous diseases manifest overlapping symptoms, rendering them arduous to differentiate. Conventional approaches predominantly depend on manual recognition by agricultural experts, a process that can be both labor-intensive and susceptible to inaccuracies. Consequently, the development of an accurate and efficient system for detecting corn leaf diseases is a pressing necessity.

In recent years, machine learning technology has been widely used in corn disease classification. Ubaidillah A et al. [1] used random forest, an artificial neural network, and a naive Bayesian algorithm to construct a four-classification model of corn diseases, and the classification accuracy was 74.44%. Panda Panigrahi K et al. [2] employed supervised machine learning techniques, such as Naive Bayes, Decision Tree, K-Nearest Neighbor, Support Vector Machine, and Random Forest, for corn plant disease detection using plant images; the Random Forest algorithm achieved the highest accuracy of 79.23% compared to other classification techniques, which could aid farmers in the early detection and classification of new image diseases as a preventive measure. Daneshwari Ashok Noola et al. [3] constructed a pertinent feature selection model using the machine learning technique with an efficient spot-tagging model, which achieved good results compared with traditional algorithms. Mishra S et al. [4] developed a real-time corn leaf disease recognition method based on a deep convolutional neural network, achieving an accuracy of 88.46%. Wang Guowei et al. [5] improved the ResNeXt101 model, and the accuracy of corn disease recognition was 89.67%, which is 0.98% higher than the original model. Although many good models have been applied to the field of corn disease monitoring, most of them have not really applied to end-to-end software, which is still in the theoretical stage.

**Figure 1:**
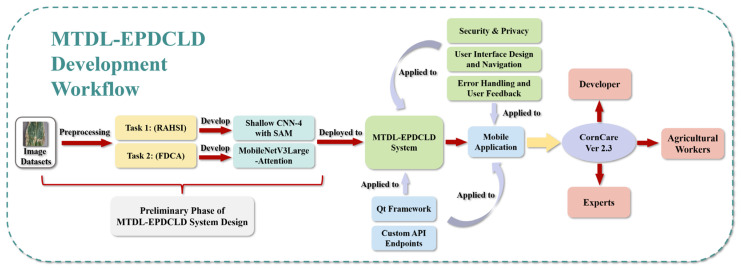
The development workflow of MTDL-EPDCLD.

To overcome these limitations, we propose a multi-task deep-learning-based system, denoted as MTDL-EPDCLD, designed to augment precision in the detection and diagnosis of corn leaf diseases. The development workflow of MTDL-EPDCLD is illustrated in Figure 1.

As shown in Figure 1, our system comprises two tasks: (1) a customized Shallow CNN-4 model incorporating an attention mechanism for the swift and accurate discernment of healthy and diseased corn leaves, and (2) a MobilenetV3Large-Attention model employed to classify the specific disease type afflicting infected leaves. To corroborate the efficacy of our proposed models, we conduct a comparative analysis with contemporary deep learning techniques. Furthermore, based on this system, we developed and implemented a mobile application that facilitates real-time, on-site disease detection and diagnosis for agricultural practitioners, which is expected to provide assistance for agricultural workers.

The highlights of our work include:

1. The formulation of a multi-task deep learning system tailored for the detection and diagnosis of corn leaf diseases;
2. The introduction of task-specific deep learning models, including the Shallow CNN-4 model with an attention mechanism for disease identification and the Mo-bilenetV3Large-Attention model for disease classification;
3. The implementation of a mobile application, enabling real-time disease detection and diagnosis to aid agricultural workers.

## 2. Materials and Methods

### 2.1. Data Source

This study employs an open-source dataset of corn leaf diseases, which was generated by amalgamating the widely recognized PlantVillage [6] and PlantDoc datasets [7]. Throughout the dataset formation process, several images were excluded as they were deemed unsuitable for the research objectives. The resulting dataset comprises RGB images depicting corn leaves afflicted with various diseases as well as healthy corn leaves. The images exhibit diverse dimensions and are categorized into the following classes in Table 1:

### 2.2. Data Preprocessing

Initially, the contrast and brightness of the images in the dataset are adjusted to emphasize image features more effectively, thereby enhancing model learning. Noise is inevitable during image processing; hence, a Gaussian filter is employed to smooth the image, reducing the impact of noise and bolstering the model’s capacity to recognize image features. Subsequently, the images are normalized using the ‘ImageDataGenerator’ function from the Keras library. This process involves rescaling pixel values by dividing them by 255 (as illustrated by the parameter ‘rescale = 1./255′), thereby bringing them within a range of 0 to 1. Normalizing the images is essential for ensuring efficient model convergence during training. Finally, all images are resized to a uniform dimension of 256 × 256 pixels (as indicated by the ‘target_size = (256, 256)’ parameter) using the custom ‘preprocess_input’ function, ensuring dimensional consistency throughout the entire dataset.

To enhance the diversity of the training data and bolster the model’s generalization capability, various data augmentation techniques are employed on the images using the ‘ImageDataGenerator’ function from the Keras library. These techniques have been proven to be effective in improving the performance of deep learning models in numerous studies [8,9]. Specifically, small horizontal and vertical translations are applied to the images, as specified by the ‘height_shift_range = [−0.005, 0, 0.005]’ and ‘width_shift_range = [−0.005, 0, 0.005]’ parameters, enabling the model to learn invariant features under translation. Random rotations are introduced with the ‘rotation_range = 10′ parameter to facilitate the model’s recognition of objects in various orientations. Additionally, horizontal flipping is performed using the ‘horizontal_flip = True’ parameter. These data augmentation techniques serve to artificially expand the dataset by generating new, transformed images based on the original ones. By training the model on this augmented dataset, the objective is to improve its ability to generalize effectively to unseen data, thereby enhancing its overall performance.

### 2.3. Model Architecture

#### 2.3.1. Task 1: Rapid and Accurate Health Status Identification (RAHSI)

A convolutional neural network (CNN) is a deep learning model with widespread application in computer vision, natural language processing, and other domains. The fundamental structure of traditional CNN consists of an input layer, convolution layers, activation functions, pooling layers, and fully connected layers. Different kinds of CNNs have been employed to classify the diseases of various plants [10,11,12,13,14,15].

**Figure 2:**
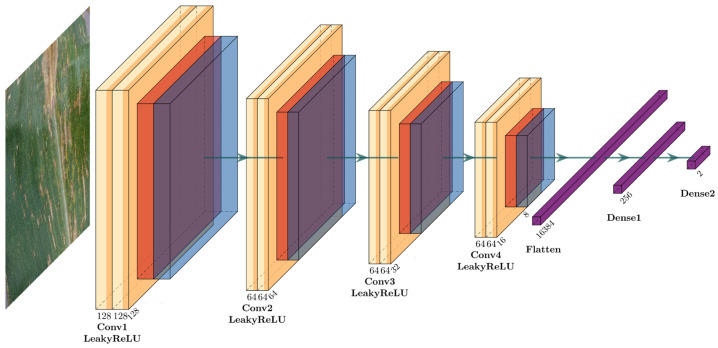
Network architecture of the proposed CNN-4 for Task 1 (RAHSI).

For the first task of rapidly and accurately identifying the health status of corn leaves, a customized shallow convolutional neural network (CNN) with spatial attention mechanism (SAM), referred to as Shallow CNN-4, is designed. This model aims to effectively and efficiently discriminate between healthy and diseased corn leaves, providing a solid foundation for the subsequent disease classification task. The network architecture of the proposed CNN-4 for Task 1 (RAHSI) is shown in Figure 2.

The architecture of Shallow CNN-4 begins with an input layer consisting of a convolution operation with 128 filters of size 3 × 3, applying the LeakyReLU activation function and employing padding set to ‘same’. The input layer can be represented as:

(1) $${Conv}_{Input}\left(input,128,3\times 3\right).$$

Following the convolution operation, a customized spatial attention layer (SAL) specifically designed for the CNN-4 model is introduced to augment the model’s focus on local features crucial for accurate classification. The SAL architecture comprises a single convolutional layer with a 1 × 1 kernel size, the sigmoid activation function, and ‘same’ padding, which can be represented as:

(2) $$SAL\left(conv,1\times 1,sigmoid\right).$$

This convolutional layer generates an attention map, which contains attention weights between 0 and 1 for each corresponding spatial region in the input feature maps. The core principle behind the SAL is to modulate the input feature maps using the computed attention weights, emphasizing the spatial regions of greatest relevance for the classification task. This is achieved through an element-wise multiplication of the attention map and the input feature maps, resulting in a new set of feature maps adjusted based on the attention weights. By incorporating the SAL into the model architecture, an improvement in classification performance is expected, as the model can better concentrate on the most informative spatial regions.

Subsequently, a max-pooling layer is applied to reduce the spatial dimensions of the feature maps, which assists in decreasing computational complexity while preserving essential features. The max-pooling layer can be represented as:

(3) $$MaxPooling\left(SAL\right).$$

To improve the model’s generalization capabilities and convergence speed, a batch normalization layer is added after the max-pooling layer, which can be represented as:

(4) $$BatchNorm\left(MaxPooling\right).$$

The combination of a convolutional layer, spatial attention layer, max-pooling layer, and batch normalization layer is repeated four times. Each of these repetitions employs 64 filters of size 3 × 3 in the convolutional layers with the LeakyReLU activation function, which can be represented as:

(5) $${Conv}_{i}\left({BatchNorm}_{i-1},64,3\times 3,LeakyReLU\right).$$

Once the four structures are stacked, the feature maps are converted into a one-dimensional vector using a flatten layer. This vector is then passed through a dense (fully connected) layer with 256 neurons and the LeakyReLU activation function. Another batch normalization layer is integrated to further enhance the model’s generalization capabilities and convergence speed. To prevent overfitting, a dropout layer with a dropout rate of 0.5 is incorporated into the model. Finally, the architecture concludes with a dense layer containing two neurons, which employs the softmax activation function to output the probability of each class—healthy or diseased corn leaves.

#### 2.3.2. Task 2: Fine-Grained Disease Classification with Attention (FDCA)

The MobileNet serial models are based on a streamlined architecture and use deep separable convolution. They are distinguished by a small number of parameters, fast calculation speed, and good accuracy, which have a wide range of applications in the classification of plant diseases [16,17,18,19].

**Figure 3:**
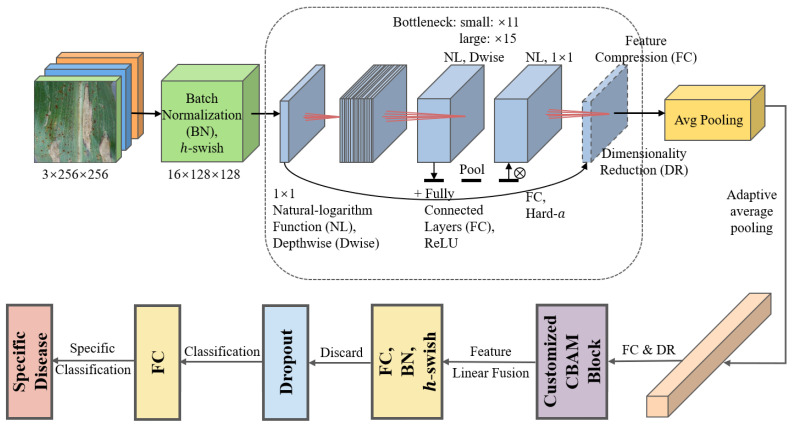
Architecture of the proposed MobileNetV3-Large model for Task 2 (FDCA).

In the second task of this study, a customized MobileNetV3Large-Attention model is proposed for the classification of specific corn leaf disease types. The architecture of the proposed MobileNetV3-Large model for Task 2 (FDCA) is shown in Figure 3.

As shown in Figure 3, this architecture builds on the standard MobileNetV3Large model, incorporating several enhancements to improve its performance in the given classification task. The enhancements are introduced in following part.

Firstly, a Convolutional Block Attention Module (CBAM) is integrated into the model to enable adaptive learning of global contextual information. The module consists of two main components: channel attention and spatial attention. The channel attention component computes the average-pooled and max-pooled features across the spatial dimensions using the following equations:

(6) $$M_{c}=\sigma \left(f_{MLP}\left(\frac{1}{H\times W}\sum_{i=1}^{H}\sum_{j=1}^{W}f_{avgpool}\left(X_{ij}\right)\right)+g_{MLP}\left(\frac{1}{H\times W}{max}_{i=1}^{H}{max}_{j=1}^{W}f_{maxpool}\left(X_{ij}\right)\right)\right)$$

where $X_{ij}$ represents the input feature maps, H and W represent the height and width of the feature maps, $f_{avgpool}$ and $f_{maxpool}$ denote the average-pooling and max-pooling operations, $f_{MLP}$ and $g_{MLP}$ are the two-layer MLPs, and σ represents the sigmoid activation function. The channel attention map $M_{c}$ is obtained by element-wise addition and serves as a weighting factor for the input feature maps, as follows:

(7) $$Y_{c}=M_{c}⊙X,$$

where $Y_{c}$ represents the channel-refined feature maps and ⊙ denotes element-wise multiplication.

The spatial attention component computes the spatial attention map using a 2D convolution layer with a 7 × 7 kernel and a sigmoid activation function, as shown in the following equation:

(8) $$M_{s}=\sigma \left(f_{conv}\left(X\right)\right),$$

where X represents the channel-refined feature maps and $M_{s}$ denotes the spatial attention map. The final refined feature maps Y are obtained by element-wise multiplication of the channel-refined feature maps and the spatial attention map:

(9) $$Y=M_{s}⊙Y_{c}.$$

Through the aforementioned method, both the average-pooled and max-pooled features undergo processing through a shared two-layer MLP (Multilayer Perceptron) with dense layers. This reduces the number of channels by the reduction ratio before restoring them to their original count. The outputs of these MLPs are combined to form the final channel attention map, which is then element-wise multiplied with the input tensor to generate the channel-refined feature maps. The spatial attention component calculates the spatial attention map by applying a 2D convolution layer with a 7 × 7 kernel and a sigmoid activation function to the channel-refined feature maps. Consequently, a single-channel spatial attention map is produced, which is element-wise multiplied with the channel-refined feature maps to yield the final refined feature maps. The CBAM returns the refined feature maps containing both channel and spatial attention information, enabling the model to concentrate on significant regions within the input images for the specific classification task.

Secondly, the learning rate is dynamically adjusted during the training process using the ReduceLROnPlateau callback. This strategy monitors the validation accuracy and reduces the learning rate when improvement in accuracy stagnates, ensuring an appropriate learning rate for the optimization process.

Additionally, an early stopping strategy based on validation accuracy is employed. This technique halts the training process when the validation accuracy surpasses a predefined threshold, preventing overfitting and reducing computational cost.

Finally, before the output layer, a dropout layer with a dropout rate of 0.5 is added to improve the model’s generalization capabilities by randomly dropping out a portion of the neurons during training. A batch normalization (BN) layer is also included to normalize the input to the output layer, which helps accelerate the training process and stabilize the model’s convergence.

#### 2.3.3. Other Mainstream Deep Learning Models for Comparison

To demonstrate the superiority of the proposed MobileNetV3Large-Attention model in the second task, its performance is compared with the unmodified MobileNetV3Large model and other mainstream deep learning models, including InceptionV3, ResNet50, DenseNet121, DenseNet169, DenseNet201, MobileNet, MobileNetV2, and MobileNetV3Small.

InceptionV3, proposed by Szegedy et al. [20], is a deep convolutional neural network that employs inception modules to efficiently capture spatial and channel-wise correlations, enabling better scaling of network depth and width. ResNet50, introduced by He et al. [21], is a 50-layer residual network that uses shortcut connections to alleviate the vanishing gradient problem, enabling the training of very deep networks without compromising performance. DenseNet121, DenseNet169, and DenseNet201, proposed by Huang et al. [22], are deep convolutional neural networks that employ dense connections between layers, ensuring efficient gradient flow and feature reuse, which lead to improved parameter efficiency and better model compactness. MobileNet, introduced by Howard et al. [23], is a lightweight deep learning model designed for mobile and embedded applications. It uses depthwise separable convolutions to reduce the number of parameters and computational complexity, making it suitable for resource-constrained environments. MobileNetV2, proposed by Sandler et al. [24], builds upon MobileNet by incorporating inverted residual blocks and linear bottlenecks, enabling the construction of efficient deep networks with reduced computational costs. MobileNetV3Large and MobileNetV3-Small, introduced by Howard et al. [25], are the latest versions of the MobileNet architecture, employing a search-based approach to optimize the network architecture for both high-performance and low-power applications.

Through the comparison with these established deep learning models, the goal is to showcase the effectiveness of the proposed MobileNetV3Large-Attention model in addressing the task of corn leaf disease detection and diagnosis. The customizations and enhancements to the MobileNetV3Large model contribute to improved performance, demonstrating the potential of this approach for real-world applications in the field of precision agriculture and plant disease management.

### 2.4. MTDL-EPDCLD System Design

The proposed MTDL-EPDCLD is a two-stage system designed to efficiently and accurately detect and diagnose corn leaf diseases. The MTDL-EPDCLD system comprises two interconnected tasks, which form a pipeline for processing input images of corn leaves. The first task, Disease Detection (Task 1), is responsible for identifying whether a given corn leaf is healthy or diseased. The second task, Disease Diagnosis (Task 2), focuses on determining the specific disease affecting a corn leaf that has been classified as diseased in the previous task. This two-stage design enables efficient processing of the input data, as only the diseased leaves are subjected to further analysis in Task 2, thereby reducing the computational complexity and resource usage of the system. To extend the functionality of the MTDL-EPDCLD system and facilitate its deployment in real-world scenarios, a mobile application has been developed that allows users to access the system’s capabilities through their smartphones or tablets. The mobile application has been designed using the Qt framework, which is a cross-platform C++ framework mainly used to develop graphical user interface applications. In this section, the design principles, software components, and development process employed to create the mobile application are outlined.

The design process for the mobile application consists of several stages, including requirement analysis, user interface (UI) design, implementation, and testing. In the requirement analysis stage, the core functionality and user interactions necessary to provide a seamless experience for users when accessing the MTDL-EPDCLD system via their mobile devices are identified. This process involves close collaboration with domain experts to ensure that the application meets the specific needs of the target users. Following the requirement analysis, the UI design is undertaken, employing the Qt Quick framework to create a responsive and user-friendly interface. Qt Quick facilitates the rapid development of fluid and dynamic UI components using QML, a declarative language that simplifies the design process and enables the efficient creation of platform-independent UI elements. With the UI design completed, the implementation phase is initiated, during which the MTDL-EPDCLD system is integrated into the mobile application. This integration involves the creation of custom API endpoints to facilitate communication between the mobile application and the deep learning models, as well as the implementation of necessary data processing and model inference functionalities within the application. Finally, thorough testing of the mobile application is conducted to ensure its reliability, performance, and compatibility across various mobile platforms. This testing process includes unit testing, integration testing, and user acceptance testing, with feedback from users and domain experts incorporated into the final design.

The mobile application provides users with an intuitive interface to access the MTDL-EPDCLD system’s capabilities. Users can capture or upload images of corn leaves, which are then processed by the system to detect and diagnose diseases. Results are displayed in an easily understandable format, with additional information on the detected diseases and potential treatment options available for reference. The application also allows users to track the history of their scans, facilitating efficient monitoring and management of corn leaf diseases.

**Figure 4:**
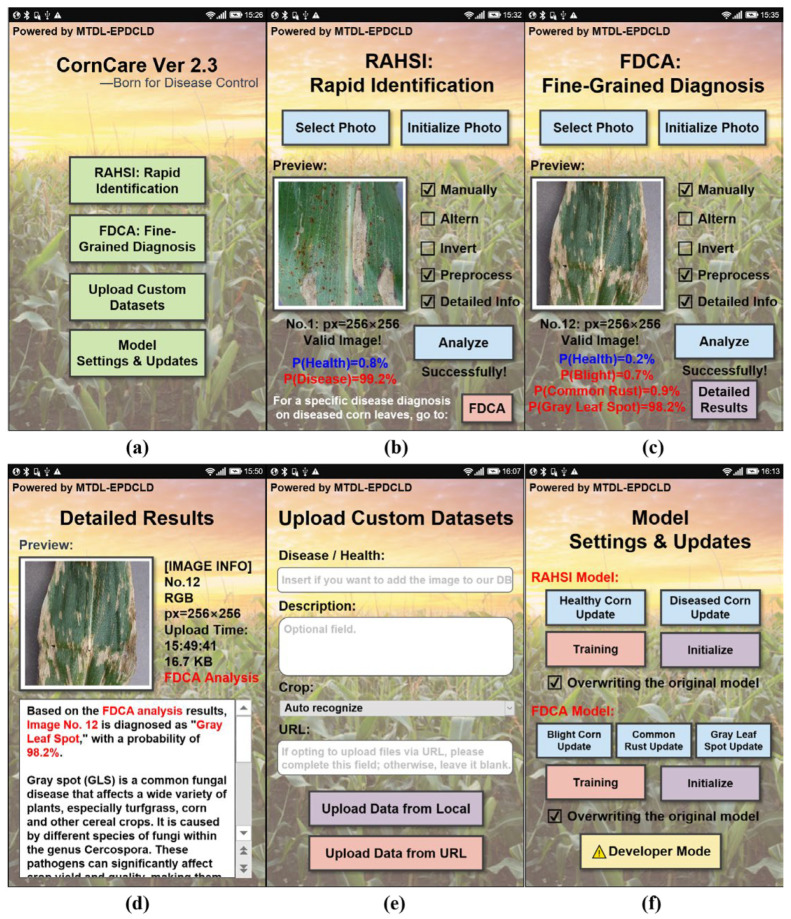
Application pages: (**a**) home page prior to image analysis, (**b**) RAHSI: Rapid Identification page, (**c**) FDCA: Fine-Grained Diagnosis page, (**d**) FDCA detailed results page, (**e**) user-uploaded custom dataset page, and (**f**) model settings and updates page.

The UI interface is shown in Figure 4, and the specific functions of the application are explained in the following part.

CornCare’s home screen (Interface 1, shown in Figure 4a) offers four buttons, providing access to the following functionalities: RAHSI: Rapid Identification, FDCA: Fine-Grained Diagnosis, Upload Custom Datasets, and Model Settings and Updates.

Interface 2, shown in Figure 4b, focuses on RAHSI: Rapid Identification, employing the Shallow CNN-4 with a spatial attention mechanism from Task 1 (RAHSI). Users can either select images from their devices or capture new images, which are then displayed with detailed information. After selecting the desired configurations, the “Analyze” button initiates rapid identification of the corn leaf’s health status. Upon successful analysis, the interface displays the probabilities of the leaf being healthy or diseased. RAHSI is designed for quick health status assessment, while FDCA provides a more detailed diagnosis of diseased leaves, albeit with a longer processing time.

Interface 3, shown in Figure 4c, is dedicated to FDCA: Fine-Grained Diagnosis, utilizing the customized MobileNetV3Large-Attention model from Task 2 (FDCA). Like Interface 2, users can select or capture images for analysis. Pressing the “Analyze” button displays the probabilities of various diseases affecting the leaf. Detailed results can be viewed by pressing the “Detailed Results” button.

Interface 4, shown in Figure 4d, shows the detailed results of FDCA, with the upper half displaying image preview and information, and the lower half containing a scrolling text window that provides diagnostic information, disease symptoms, and prevention and treatment recommendations.

Interface 5, shown in Figure 4e, enables users to upload custom datasets, which are incorporated into the system’s DB database. Files can be uploaded either locally or via URL. The app ensures a secure and efficient process from file selection to storage on the server.

Interface 6, shown in Figure 4f, offers model settings and updates, allowing users to configure and update both the RAHSI and FDCA models. A developer mode, accessible with a pre-set password, grants access to advanced features and debugging tools for maintaining device stability and security.

In summary, the CornCare mobile application provides an intuitive design, comprehensive functionality, and high-performance models for corn leaf disease detection and diagnosis. Its efficient and streamlined processes make it an invaluable asset in agricultural scenarios, contributing to the development of more effective disease management strategies.

### 2.5. Experimental Setup

All experiments were conducted using an Intel(R) Xeon Gold 6142 @ 2.6 GHz CPU, 27.1 GB RAM, and NVidia GeForce RTX 3080 GPU. The experimental environment involved Docker v20.10.10, Python v3.7, PyTorch v1.10, and TensorFlow v2.7.0. The MTDL-EPDCLD system was designed as a multi-task framework, where Task 1 (RAHSI) was performed first, followed by Task 2 (FDCA).

In Task 1 (RAHSI), ‘sparse_categorical_crossentropy’ was utilized as the loss function, and the Adam optimizer was employed to update the weights. To expedite the identification of optimal model performance during the training process, the advantages of grid search and random search were combined, and the Grid-Search with Random Sampling approach was implemented for parameter optimization. The dataset was partitioned into training and testing sets with a 70:30 ratio, using a batch size of 32 and training for a total of 80 epochs. Data splitting was performed using the ‘train_test_split function’ from the Scikit-learn library, with a random state of 314 to ensure consistent results across multiple runs.

In Task 2, the same loss function, ‘sparse categorical crossentropy’, was employed, and the Adam optimizer was utilized for weight updates. To expedite the identification of optimal model performance during training, the advantages of grid search and random search were combined, implementing a Grid-Search with Random Sampling approach for parameter optimization. The dataset was partitioned into training and validation sets at a 70:30 ratio using Keras’ ImageDataGenerator. Both sets were generated using the flow_from_directory method, with data shuffled. The batch size was set to 32, and the training process spanned 50 epochs. The number of steps per epoch for training and validation was determined by the length of the respective data generators. Additionally, the ‘ReduceLROnPlateau’ callback was employed to monitor and adjust the learning rate during training, and a customized early stopping callback based on validation accuracy was used.

### 2.6. Experimental Evaluation

In this study, the effectiveness of the trained classification model is evaluated using various performance metrics, including Accuracy, Loss, Precision, Recall, F1 score, and area under the receiver operating characteristic (ROC) curve. The evaluation metrics are calculated using the following equations:

(10) $$Accuracy=\frac{TP+TN}{TN+FP+FN+TP},$$

(11) $$Precision=\frac{TP}{TP+FP},$$

(12) $$Recall=\frac{TP}{TP+FN},$$

(13) $$F_{1}=\frac{2\times Precision\times Recall}{Precision+Recall},$$

where TP (True Positive) denotes the number of positive samples correctly classified into the class by the network model; FP (False Positive) denotes the number of negative samples incorrectly classified as positive by the network model; FN (False Negative) denotes the number of positive samples incorrectly classified as negative by the network model; TN (True Negative) indicates the number of negative samples correctly classified as negative by the network model. The F_1_ value is a measure of the network model’s performance that combines its recall and accuracy into a single value. Specifically, it represents the weighted average of the two metrics.

On the other hand, the AUC area refers to the area under the ROC curve, which is a graphical representation of the model’s performance. The ROC curve plots the True-Positive Rate (TPR) against the False-Positive Rate (FPR) of the classifier at various decision thresholds. This relationship is calculated using Equations (14) and (15), which allow for assessing the performance of the model across different thresholds.

(14) $$TPR=\frac{TP}{TP+FN},$$

(15) $$FPR=\frac{FP}{FP+TN}.$$

The AUC ranges from 0 to 1, with higher values indicating better performance. As the AUC value approaches 1, it suggests that the classifier has a better ability to distinguish between positive and negative instances. In other words, a higher AUC implies that the model has a greater ability to correctly classify instances and assign higher scores to positive instances than negative instances.

## 3. Results

### 3.1. Visualization of Preprocessing Results

**Figure 5:**
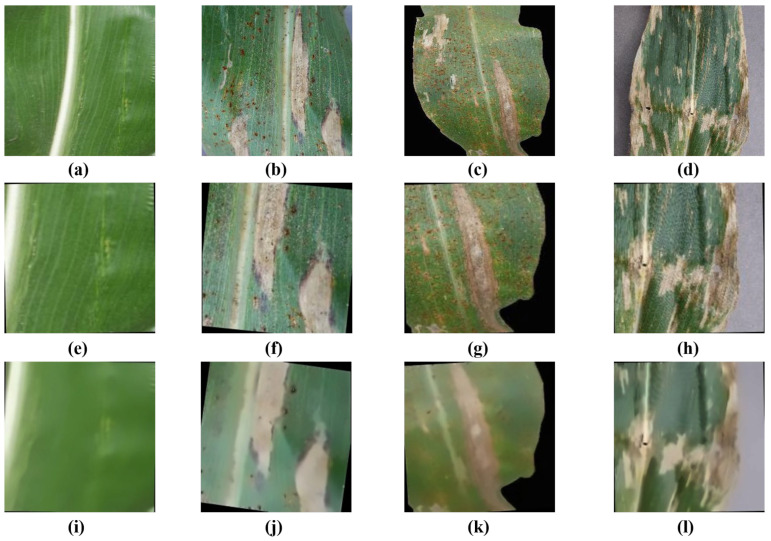
Example images and preprocessed representations of corn leaf diseases: (**a**) sample images of health, (**b**) sample images of blight, (**c**) sample images of common rust, (**d**) sample images of gray spot, (**e**) augmented images of health, (**f**) augmented images of blight, (**g**) augmented images of common rust, (**h**) augmented images of gray spot, (**i**) denoised images of health, (**j**) denoised images of blight, (**k**) denoised images of common rust, and (**l**) denoised images of gray spot.

The corn disease dataset used in this paper contains 4187 images, including leaf samples with blight, common rust, gray spot, and healthy leaf samples, as shown in Figure 5a–d. The original images are processed using data enhancement, and the results are presented in Figure 5e–h. Then, the images are smoothed with Gaussian filtering to get the ones presented in Figure 5i–l.

### 3.2. Results of Task 1 (RAHSI) Model

In Task 1, the model is compiled using ‘sparse categorical crossentropy’ as the loss function and the Adam optimizer for weight updates. The optimal learning rate, batch size, and other hyperparameters are chosen to maximize model performance. The model undergoes training for 80 epochs with a batch size of 32, employing a ‘ReduceLROnPlateau’ callback to automatically adjust the learning rate based on validation loss. The training process is timed to evaluate computational efficiency.

The performance of various CNN architectures (CNN-1, CNN-2, CNN-3, CNN-4, and CNN-5) was evaluated, and CNN-4 demonstrated the best performance, indicating that a shallow multi-layer CNN-4 architecture with limited depth is most suitable for this binary classification task.

**Figure 6:**
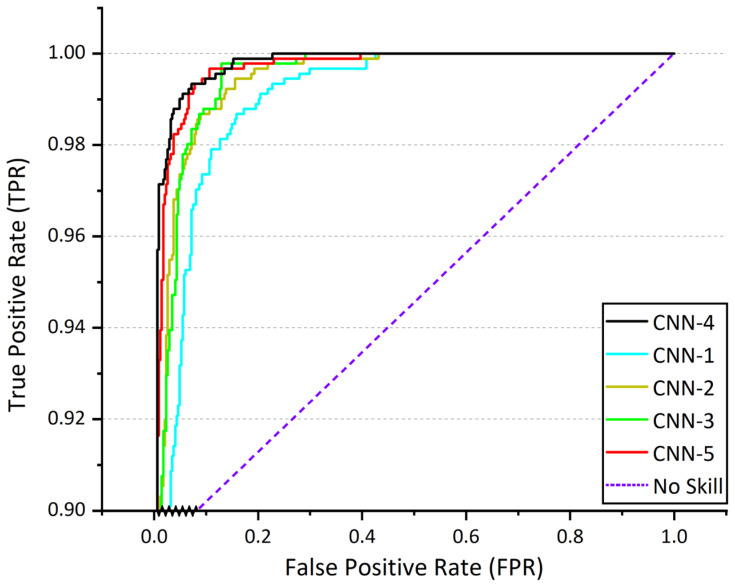
ROC curves of various CNN architectures (CNN-1, CNN-2, CNN-3, CNN-4, and CNN-5).

The results obtained from Task 1 (RAHSI)’s model (CNN-4) demonstrate exceptional performance across various evaluation metrics. The model attains an accuracy of 0.9999 on the training set and 0.9873 on the validation set. The loss values for the training and validation sets amount to 0.003 and 0.06, respectively. The model’s precision, recall, and F1 score, calculated using a macro-average, reach 0.98, 0.99, and 0.98, respectively, indicating a high degree of consistency in classifying different categories. Moreover, the model achieves an AUC score of 0.9985, reflecting its robust discriminative capacity between healthy and diseased corn leaves. The results emphasize the effectiveness of the proposed model in swiftly and accurately determining the health status of corn leaves. Figure 6 shows ROC curves of various CNN architectures (CNN-1, CNN-2, CNN-3, CNN-4, and CNN-5). Table 2 shows the values of the evaluation metrics for Task 1 (RAHSI) model and various CNN models.

### 3.3. Results of Task 2 (FDCA) Model

#### 3.3.1. Results of the Customized MobileNetV3Large-Attention Model

The objective of Task 2 is to develop a model capable of fine-grained disease classification with attention, focusing on the accurate identification and discrimination of various corn leaf diseases. The results obtained from the model for Task 2 demonstrate superior performance across multiple evaluation metrics. Considering the imbalance in the number of samples in different categories, we adopt macro-averaging to calculate precision, recall, and F1 score, ensuring the accuracy of these evaluation metrics. The model achieves an accuracy of 1.0000 on the training set and 0.9444 on the validation set. The loss values for the training and validation sets are 0.0009 and 0.3149, respectively. For the macro-average calculations, the model’s precision, recall, and F1 score are 0.9293, 0.9369, and 0.9328, respectively, indicating a high degree of consistency in the classification of different corn leaf disease classes. Furthermore, the model achieves an AUC score of 0.9993, signifying its strong discriminative ability between various corn leaf diseases.

The results highlight the effectiveness of the proposed model in achieving the goal of fine-grained disease classification with attention, providing valuable insights for accurate diagnosis and intervention. The performance metrics showcase the model’s ability to overcome the challenges posed by class imbalance, demonstrating its potential in real-world applications for corn leaf disease detection and diagnosis.

#### 3.3.2. Comparison of Task 2 (FDCA) Model with Other Mainstream Models

In Task 2 (FDCA), the superiority of the proposed MobileNetV3Large-Attention model for fine-grained disease classification is demonstrated by comparing its performance with the unmodified MobileNetV3Large model and other mainstream deep learning models, including InceptionV3, ResNet50, DenseNet121, DenseNet169, DenseNet201, MobileNet, MobileNetV2, and MobileNetV3Small.

The MobileNetV3Large-Attention model achieves the highest validation accuracy of 94.44%, significantly outperforming the other models. In comparison, the unmodified MobileNetV3Large model obtains a validation accuracy of 91.92%. The other deep learning models, such as InceptionV3, ResNet50, and DenseNet series, have validation accuracies ranging from 87.55% to 91.26%, indicating a clear advantage in classification performance for the proposed model.

Regarding other evaluation metrics, the MobileNetV3Large-Attention model also delivers impressive results. It exhibits the lowest validation loss (0.3149), the highest precision (0.92926), recall (0.9369), F1 score (0.93283), and AUC (0.99932072). These results further demonstrate the effectiveness of the attention mechanism incorporated in the model for fine-grained disease classification, allowing for superior performance compared to the other models tested.

**Figure 7:**
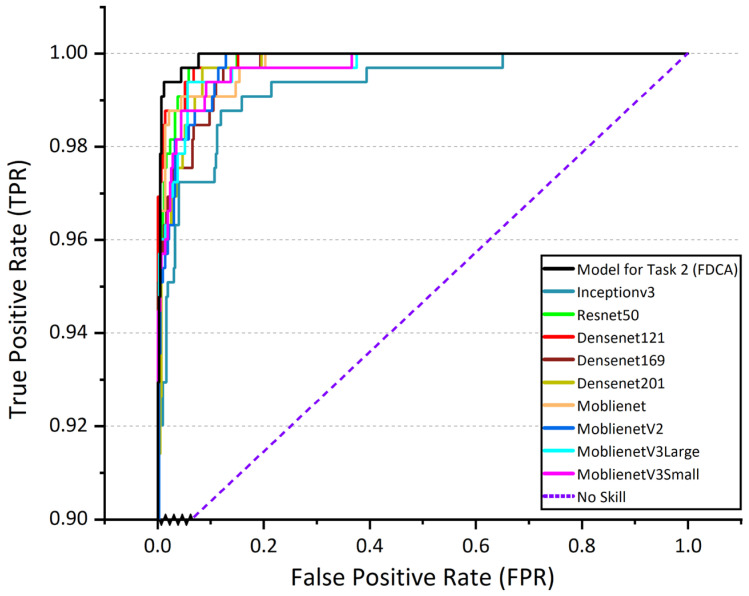
ROC curves of Task 2 (FDCA) model and other mainstream deep learning models.

In summary, the proposed MobileNetV3Large-Attention model exhibits outstanding performance in Task 2 (FDCA), outperforming other mainstream deep learning models and providing a reliable solution for fine-grained corn leaf disease classification in real-world agricultural conditions. Figure 7 shows ROC curves of Task 2 (FDCA) model and other mainstream deep learning models. Table 3 shows the values of the evaluation metrics for Task 2 (FDCA) model and other mainstream deep learning models.

## 4. Discussion

In this study, a Multi-Task Deep Learning-based system (MTDL-EPDCLD) was developed for enhanced precision detection and diagnosis of corn leaf diseases. The system was designed to perform two main tasks: Task 1 (RAHSI) for rapid identification of healthy and diseased leaves, and Task 2 (FDCA) for fine-grained diagnosis of specific diseases. Task 1 employed the Shallow CNN-4 with a spatial attention mechanism, while Task 2 utilized the customized MobileNetV3Large-Attention model. By comparing these models with several mainstream deep learning models, it was demonstrated that they outperformed others in terms of accuracy, loss, precision, recall, F1 score, and AUC.

The selection of the Shallow CNN-4 with a spatial attention mechanism for Task 1 was based on preliminary experiments, which indicated that a shallow CNN architecture with a limited number of layers was most suitable for this binary classification task. The performance of different CNN architectures (CNN-1, CNN-2, CNN-3, CNN-4, and CNN-5) was evaluated, and CNN-4 achieved the best performance, confirming its suitability for rapid identification of corn leaf health status.

For Task 2, a customized MobileNetV3Large-Attention model was selected based on preliminary experiments comparing it with other mainstream models. The model achieves excellent results due to several key improvements, such as the addition of an attention mechanism, which significantly enhances performance metrics and addresses fine-grained diagnostic tasks in a more focused manner. The ReduceLROnPlateau callback rate is used to dynamically adjust the learning rate, employing a strategy that monitors validation accuracy and reduces the learning rate when accuracy improvement stalls, thereby ensuring an appropriate learning rate for the optimization process. Furthermore, an early stopping strategy based on validation accuracy is adopted. This technique halts the training process when the validation accuracy exceeds a predefined threshold, preventing overfitting and reducing computational costs.

The MTDL-EPDCLD system carries substantial importance for future agricultural production, as corn, a major staple crop globally, faces significant threats from corn leaf diseases. Prompt and precise detection and diagnosis of these diseases are crucial for implementing effective control measures and minimizing crop losses. The system provides a robust tool for farmers and agricultural experts to detect and diagnose corn leaf diseases with improved accuracy, enabling rapid identification of healthy and diseased leaves and detailed disease classification. This empowers farmers to make informed decisions on disease management strategies, allowing for early intervention, targeted treatments, and optimized resource allocation, significantly mitigating disease impact on corn crops and improving overall crop yield and quality. The system’s implications for agricultural production include promoting site-specific disease control measures, reducing pesticide use, minimizing environmental impact, and facilitating integrated pest management strategies. Early detection and diagnosis enable farmers to implement preventive measures and use disease-resistant crop varieties, decreasing chemical interventions’ reliance and fostering ecological balance. Moreover, the MTDL-EPDCLD system’s enhanced precision detection and diagnosis can potentially increase crop yields and agricultural productivity. By effectively managing corn leaf diseases, farmers can minimize yield losses due to infections, ensuring a stable and abundant corn supply, crucial for global food security and agricultural communities’ well-being. In conclusion, the MTDL-EPDCLD system offers significant value to agricultural production by enabling accurate and timely corn leaf disease detection and diagnosis, empowering farmers to protect their crops and optimize agricultural practices. The system contributes to increased crop yields, reduced environmental impact, and sustainable agriculture, representing a promising advancement in precision agriculture with the potential to transform disease control strategies and positively impact global food security.

Despite its strengths, this study has some limitations. First, the dataset used in the study is relatively small, which may affect the generalizability of the results. Future research could benefit from incorporating larger and more diverse external datasets. Second, the system’s functionalities could be further refined and expanded to address a broader range of agricultural challenges. Finally, the training cost of the models could be further reduced to make the system more accessible and practical for real-world applications.

## 5. Conclusions

In conclusion, this study presents the development of a multi-task deep-learning-based system, MTDL-EPDCLD, for enhanced accurate detection and diagnosis of corn leaf diseases. The approach involves two tasks: Task 1 (RAHSI) rapidly identifies healthy and diseased corn leaves using a shallow CNN-4 model with a spatial attention mechanism, and Task 2 (FDCA) employs a customized MobileNetV3Large-Attention model for fine-grained diagnosis of specific diseases.

Experimental results demonstrate that the model for Task 1 (RAHSI) exhibits superior performance across various evaluation metrics. The model attains an accuracy of 0.9999 on the training set and 0.9873 on the validation set. The loss values for the training and validation sets are 0.003 and 0.06, respectively. The precision, recall, and F1 score of the model, calculated using a macro-average, reach 0.98, 0.99, and 0.98, respectively, indicating a high degree of consistency in classifying different categories. Moreover, the model achieves an AUC score of 0.9985, reflecting its robust discrimination between healthy and diseased corn leaves. The results emphasize the effectiveness of the proposed model in swiftly and accurately determining corn leaf health. For Task 2 (FDCA), the proposed MobileNetV3Large-Attention model outperforms several mainstream deep learning models in terms of accuracy, loss, precision, recall, F1 score, and AUC. The val_accuracy of this model is 94.44%, approximately 5% higher than other models. For precision, recall, and F1 score, the model achieves 0.92926, 0.9369, and 0.93283, respectively, indicating a 4–8% improvement over other models. Furthermore, the model attains an AUC of 0.9993, representing an increase of 0.002–0.007 compared to other mainstream deep learning models.

The MTDL-EPDCLD system holds significant potential for increasing agricultural productivity by enabling farmers to make timely and informed decisions about disease management. Consequently, this could lead to enhanced crop yields, improved food security, and a more sustainable agricultural sector. Although some limitations exist, such as the size of the dataset and the need to further refine the system’s capabilities, this research represents a crucial advancement in applying deep learning techniques to address pressing agricultural challenges.

In summary, the MTDL-EPDCLD system offers a promising solution for detecting and diagnosing corn leaf diseases, and its continued development and implementation may substantially impact agricultural practices and outcomes.

## Figures and Tables

**Table 1:** Dataset description.

Label	Disease	Number of Images
0	Common Rust	1306
1	Gray Leaf Spot	574
2	Blight	1146
3	Healthy	1162

**Table 2:** The values of the evaluation metrics for Task 1 (RAHSI) model and various CNN models.

Model	Acc	Val_Acc	Loss	Val_Loss	Precision	Recall	F1-Score	AUC
CNN-5	0.9997	0.9801	0.0017	0.0620	0.97	0.98	0.98	0.9981
CNN-4	1.0000	0.9873	0.0030	0.0600	0.98	0.99	0.98	0.9985
CNN-3	0.9988	0.9785	0.0020	0.0810	0.97	0.98	0.97	0.9958
CNN-2	1.0000	0.9690	0.0002	0.1343	0.97	0.97	0.97	0.9916
CNN-1	0.9920	0.9547	0.0030	0.1846	0.96	0.96	0.96	0.9872

**Table 3:** The values of the evaluation metrics for Task 2 (FDCA) model and other mainstream deep learning models.

Model	Acc	Val_Acc	Loss	Val_Loss	Precision	Recall	F1-Score	AUC
InceptionV3	0.9762	0.8755	0.1007	0.3001	0.8510	0.8530	0.8520	0.9925
ResNet50	0.9938	0.9152	0.0433	0.2566	0.8956	0.8965	0.8960	0.9982
DenseNet121	0.9621	0.9033	0.1213	0.2265	0.8756	0.8834	0.8790	0.9988
DenseNet169	0.9714	0.9113	0.0977	0.2252	0.8937	0.8846	0.8888	0.9971
DenseNet201	0.9727	0.9126	0.0874	0.2358	0.8920	0.9003	0.8958	0.9972
MobileNet	0.9797	0.9073	0.0811	0.2384	0.8839	0.8847	0.8843	0.9979
MobileNetV2	0.9789	0.8914	0.0817	0.2826	0.8651	0.8744	0.8689	0.9972
MobileNetV3Large	0.9824	0.9192	0.0621	0.2356	0.9025	0.8981	0.9000	0.9970
MobileNetV3Small	0.9806	0.9046	0.0845	0.2501	0.8812	0.8904	0.8850	0.9970
MobileNetV3Large-Attention	1.0000	0.9444	0.0009	0.3149	0.9293	0.9369	0.9328	0.9993

## Data Availability

The data presented in this study are available in this article.
